# Assessment of h-index and associated demographic and academic parameters for academic hematologists in Canada

**DOI:** 10.3389/fmed.2024.1457366

**Published:** 2024-08-30

**Authors:** Daniel Josué Guerra Ordaz, Jean Roy, Imran Ahmad, Mohammed Kaouache, Brandon Ramchatesingh, Sera Whitelaw, Anna Nikonova, Chris Bredeson, Ivan V. Litvinov

**Affiliations:** ^1^Faculty of Medicine and Health Sciences, McGill University, Montreal, QC, Canada; ^2^Département de Médecine, Université de Montréal, Montreal, QC, Canada; ^3^The Research Institute of the McGill University Health Centre, Montreal, QC, Canada; ^4^Division of Hematology, McGill University Health Centre, Montreal, QC, Canada; ^5^Ottawa Hospital Research Institute, Ottawa University, Ottawa, ON, Canada; ^6^Division of Dermatology, McGill University Health Centre, Montreal, QC, Canada

**Keywords:** hematology, h-index, sex, academia, Canada, CIHR funding, regional analysis

## Abstract

**Introduction:**

The h-index measures researchers’ productivity by assessing simultaneously the number of publications and citations. We aimed to assess the factors that could influence h-index for hematologists practicing in academic institutions in Canada.

**Methods:**

We identified universities with a hematology residency training programs/fellowships using the Canadian Resident Matching Service (CaRMS) website. We obtained the listing of faculty, sex, and academic ranks by consulting faculty directories or by contacting respective departments/universities, when directories were unavailable or incomplete. For each faculty member, we obtained years since Royal College of Physicians’ and Surgeons of Canada certification or equivalent, receipt of Canadian Institute of Health Research (CIHR) grants within the last 5 years, attainment of graduate degrees (M.Sc., Ph.D., other), and the h-index.

**Results:**

The data included information collected from 372 individuals (171 females) across Canada (Atlantic Provinces: 13; Quebec: 89; Ontario: 182; Prairie Provinces: 59; British Columbia: 29). Univariate analysis showed that male sex, practicing in British Columbia, longer duration since specialty certification, completion of an M.Sc. or a Ph.D. degree, attaining a higher academic rank and receiving CIHR funding were associated with higher h-index. The results of the univariate analysis were concordant with the multivariate analysis, except that practicing in Ontario was also associated with higher h-index.

**Conclusion:**

This study provides details on the h-index curve/parameters for academic productivity of hematologists in Canada. Importantly, based on multivariate analysis, higher h-index was associated with male sex, location of practice, years since certification, attainment of M.Sc. or Ph.D. degrees, academic rank, and recent CIHR funding.

## Introduction

Hematology is a medical subspeciality recently marked by new scientific paradigms and their direct application in routine care, mainly led by novel diagnostic and therapeutic tools in both benign and malignant diseases (e.g., next-generation gene sequencing, immune, cell and gene therapy). The role of academic hematologists in the discovery and dissemination of such advances is paramount, ultimately leading to improved survival and quality of life of patients with blood disorders.

To quantify scientific productivity, the h-index has emerged as a key standardized metric, providing a comprehensive measure of individuals’ research contributions by considering both the number of publications and the citations they receive (1, 2). It assesses researcher’s impact and influence within the academic community. A higher h-index therefore signifies a more influential body of work that continues to be acknowledged and cited by peers. The h-index is considered for hiring, promotions, awarding grants/awards by study panels and represent the most commonly used metric in academia.

It was suggested that an h-index of 12 may be typical for advancement from assistant professor to associate professor and an h-index of 18 may be typical for advancement from associate professor to professor. However, the model underlying Hirsch’s h-index is based on data from basic scientists specializing in physics and not clinician-scientists (3). The h-index from basic science may not be directly comparable to clinical research. Trends in h-index among some specialist physicians in Canada have been investigated, however, it is not well established what is considered as a favorable h-index in hematology in Canada and little is known regarding the factors impacting the h-index in this context.

We note that in addition to the h-index other metrics have been proposed that could be used in combination. As such, normalized *h-*index (4) divides the *h-*index by the number of publications produced over the years, as detailed in the equation below (4).


$$h^{n}=\frac{h}{P}$$

Some also utilize v-index, which highlights the percentage of publications reflected in the *h-*index, as shown in the equation below (5).


$$\upsilon =100h^{n}=100\frac{h}{P}$$

Finally, m-index was proposed as a measure to account for the length of an investigator’s career. Generally, longer careers enable accumulation of higher number of citations. To address this, m-index and other time-scaled indexes were proposed. To obtain an m-index, h-index is simply divided by the number of years.

In our study, we aimed to investigate factors associated with academic productivity in hematology across premier university-affiliated medical centres. We sought to define the h-index curve and identify factors that might have an impact on academic productivity/h-index as well as explore regional variations.

## Methods

For this cross-sectional study, we identified 14 universities offering a hematology fellowship/residency program through the Canadian Resident Matching Service (CaRMS) website. When available, we consulted the faculty directories for each university for respective hematology divisions to obtain the complete list of members (search conducted between November 2022 and March 2023). As the complete list of members was not available for five universities, we contacted administrators and/or Faculty members at McGill University, Université Laval, Université de Montréal, Université de Sherbrooke, and the University of Ottawa to obtain a complete list of faculty members (completed in March 2023). As this study used only publically available data, it was exempted from ethics review.

Faculty members with one of the following clinical ranks were included: lecturer/instructor, assistant professor, associate professor, and full professor. All physicians with a subspeciality diploma in internal medicine, hematology, or oncology and leading clinical practice in hematology defined as blood cancers, hemostasis, thrombosis, transfusion medicine, hematopoietic cell transplantation, and benign hematology were included. Members without an academic position, with a Ph.D. but not being a physician (MD or equivalent), retired as of January 1, 2023, rural practitioners, or physicians with primary practice in solid tumours were excluded. A single investigator extracted the data (DJGO) with validation performed by a senior investigator (JR).

Each faculty member’s full name, sex, affiliated university’s area (Atlantic (ATL), Quebec (QC), Ontario (ON), Prairie Provinces including Manitoba, Saskatchewan and Alberta (PRAIRIE), British-Columbia (BC)), rank (lecturer, assistant professor, associate professor, professor), number of years since Collège des Médecins du Québec (CMQ) and/or the Fellow of the Royal College of Physicians of Canada (FRCPC) certification, or another international equivalent certification, post-graduate studies (none, M.Sc., Ph.D., other), recent Canadian Institute of Health Research (CIHR) funding (yes/no), and their h-index (based on Scopus Author ID search, completed in March 2023) was collected (1). For this study, only biological sex information was used as gender narratives or self-reported gender information was unavailable.

The same approach was used to find faculty members for all positions. This approach was inspired by the methodology we used in our previous paper on academic dermatology (6). Our initial search used authors’ first and last names as they appeared on the faculty website and their institutional affiliation. If unsuccessful, we searched by last name and affiliation, then by last name alone and verified by evaluating published papers as being relevant to hematology in their Scopus profile. We also searched faculty listings on the department website for post-graduate degrees. If the data was not readily available, we searched LinkedIn© or Google/Google Scholar to locate research articles and websites for medical centres or conferences (4).

The CMQ directory was consulted for Quebec hematologists (June 1, 2023) to determine the number of years since certification. The FRCPC directory was consulted for non-Quebec Canadian hematologists (June 1, 2023) to determine the number of years since certification. For Canadian hematologists without FRCPC certification (e.g., hematologists who trained abroad/not certified by the FRCPC), a LinkedIn (June 1, 2023) search was performed to determine the number of years since certification. If not available, we searched on respective university websites or the College of Physicians and Surgeons for their respective provinces. For hematologists with more than 30 years since the FRCPC certification or equivalent, we searched for their names in the provincial physician directories to confirm they were still in practice.

For university affiliation, we referred to the main campus geographic location detailed on the website. We categorized them into five different geographic regions: Atlantic (Dalhousie University), Quebec (McGill University, Université de Montréal, Université de Sherbrooke, Université Laval), Ontario (McMaster University, Queen’s University, University of Ottawa, University of Toronto, Western University), Prairie Provinces (University of Alberta, University of Calgary, University of Manitoba), and British Columbia (University of British Columbia).

As CIHR is Canada’s most well-known funding organization with a publicly accessible funding database, we used this database to obtain research funding held as of June 1, 2023. Only data for the last 5 years was included. The h-index was obtained from author profiles on Scopus Author ID on June 1, 2023 (5). The highest related h-index was used when an author had numerous profiles available.

### Statistical analyses

Descriptive statistics were used, including mean and median h-index, mean and median number of years since FRCPC, CMQ, or international equivalent certification, distribution of academic rank, affiliated university geographical distribution, CIHR funding and graduate degree (M.Sc. / Ph.D.) for all individuals, by sex were reported as previously described (6). Univariate and multivariate analyses were performed to assess the association between h-index and years since FRCPC certification or equivalent, academic rank, affiliated university geographical distribution, CIHR funding, graduate degree completion, and sex using complete data (6). The univariate association of each covariate with the h-index was explored using graphical methods.

Because the h-index can only take positive values (only 3 individuals had an h-index of zero) and using it as the response variable in a linear model may lead to negative predated values, we selected to shift the h-index by one unit to the right and applied the natural log transformation. With this transformation, when we used the exponential of an estimated beta coefficient and subtracted 1, we obtained the percent change in the shifted h-index for a one-unit increase in the variable of interest while adjusting for the remaining covariates. Variables included in the model were sex, CIHR funding in the last 5 years, earned graduate (M.Sc. or Ph.D.) degree, jurisdictions of practice, years since FRCPC certification, and academic rank. The primary analysis was completed and a sensitivity analysis was used where results were pooled following multiple imputations by chained equations. Multicolinearity was assessed using the generalized variance inflation factors (GVIF) (7). For each continuous or categorical variable, GVIF^(1/(2*Df)) was used as an estimate of the decrease in precision of estimation for the variable in question due to correlations with one or more of the remaining variables (7).

## Results

In total, 546 hematology faculty members across Canada were identified, of which 174 were excluded (19 retired, 37 non-physicians, 2 non-academic positions, 68 having main practice in oncology, 36 practicing in non-academic centres, 12 without retrievable h-index). Ultimately, 372 faculty members were included, 171 females (46%; Table 1). The median number of years since the FRCPC certification was 15.0 [9.0, 24.0]. The median number of years since FRCPC certification was significantly different (*p* < 0.001) by region, with the lowest in Atlantic Canada (12.0 [8.25;21.8]) and the highest in BC (22.0 [14.0;29.5]). ON had the highest number of members holding a graduate degree (47%), while BC had the highest percentage of members holding a Ph.D. (17%) degree. Nationally, 64 members were holding a CIHR grant, with ON having 42 members representing 66% of CIHR awardees in our cohort. The median h-index for the entire cohort was 11.0 [4.0; 26.3] (mean: 18.1, SD: 19.7). We observed a significantly different median h-index within regions (*p* = 0.018), with the lowest in ATL (6.00 [2.00;15.0]) and the highest in BC (17.0 [9.00;33.0]), with intermediate values in QC (9.00 [4.00, 22.0]), PRAIRIE provinces (10.0 [4.50, 18.0]) and ON (11.0 [4.00, 31.0]).

**Table 1:** Characteristics of faculty members across hematology academic centres in Canada.

	ATL	QC	ON	PRAIRIE	BC	Canada	*p*-value
Sex	*n* (%)	*n* (%)	*n* (%)	*n* (%)	*n* (%)	*n* (%)	0.436
Female	6 (46)	42 (47)	76 (42)	30 (51)	17 (59)	171 (46)	
Male	7 (54)	47 (53)	106 (58)	29 (49)	12 (41)	201 (54)	
Median number of years since FRCPC certification [IQR]	12.0 [8.25; 21.8]	20.0 [12.0; 29.0]	13.0 [7.00; 24.0]	13.0 [8.00; 18.0]	22.0 [14.0; 29.5]	15.0 [9.0; 24.0]	**<0.001**
Graduate degree							**0.016**
None	8 (62)	70 (79)	96 (53)	39 (66)	17 (59)	230 (62)	
Other master’s degree	1 (8)	2 (2)	18 (10)	3 (5)	3 (10)	27 (8)	
M.Sc.	4 (31)	8 (9)	48 (26)	12 (20)	4 (14)	76 (20)	
Ph.D.	0 (0)	9 (10)	20 (11)	5 (8)	5 (17)	39 (11)	
Academic rank							0.059
Lecturer	0 (0)	5 (6)	23 (13)	1 (2)	3 (10)	32 (9)	
Assistant Professor	9 (69)	40 (45)	70 (39)	34 (58)	11 (38)	164 (44)	
Associate Professor	2 (15)	24 (27)	48 (26)	18 (31)	10 (35)	102 (27)	
Professor	2 (15)	20 (23)	41 (23)	6 (10)	5 (17)	74 (20)	
CIHR funding							**0.033**
No	12 (92)	78 (88)	140 (77)	50 (85)	28 (97)	308 (83)	
Yes	1 (8)	11 (12)	42 (23)	9 (15)	1 (3)	64 (17)	
Median h-index [IQR]	6.00 [2.00; 15.0]	9.00 [4.00; 22.0]	11.0 [4.00; 31.0]	10.0 [4.50; 18.0]	17.0 [9.00; 33.0]	11.0 [4.0; 26.3]	**0.018**

ATL, Atlanti region; QC, Quebec; ON, Ontario; PRAIRIE, Prairie provinces; BC, British Columbia; FRCPC, Fellow of the Royal College of Physicians of Canada or equivalent certification; M.Sc., Master of Science; Ph.D., Doctor of Philosophy; CIHR, Canadian Institutes of Health Research.

**Figure 1:**
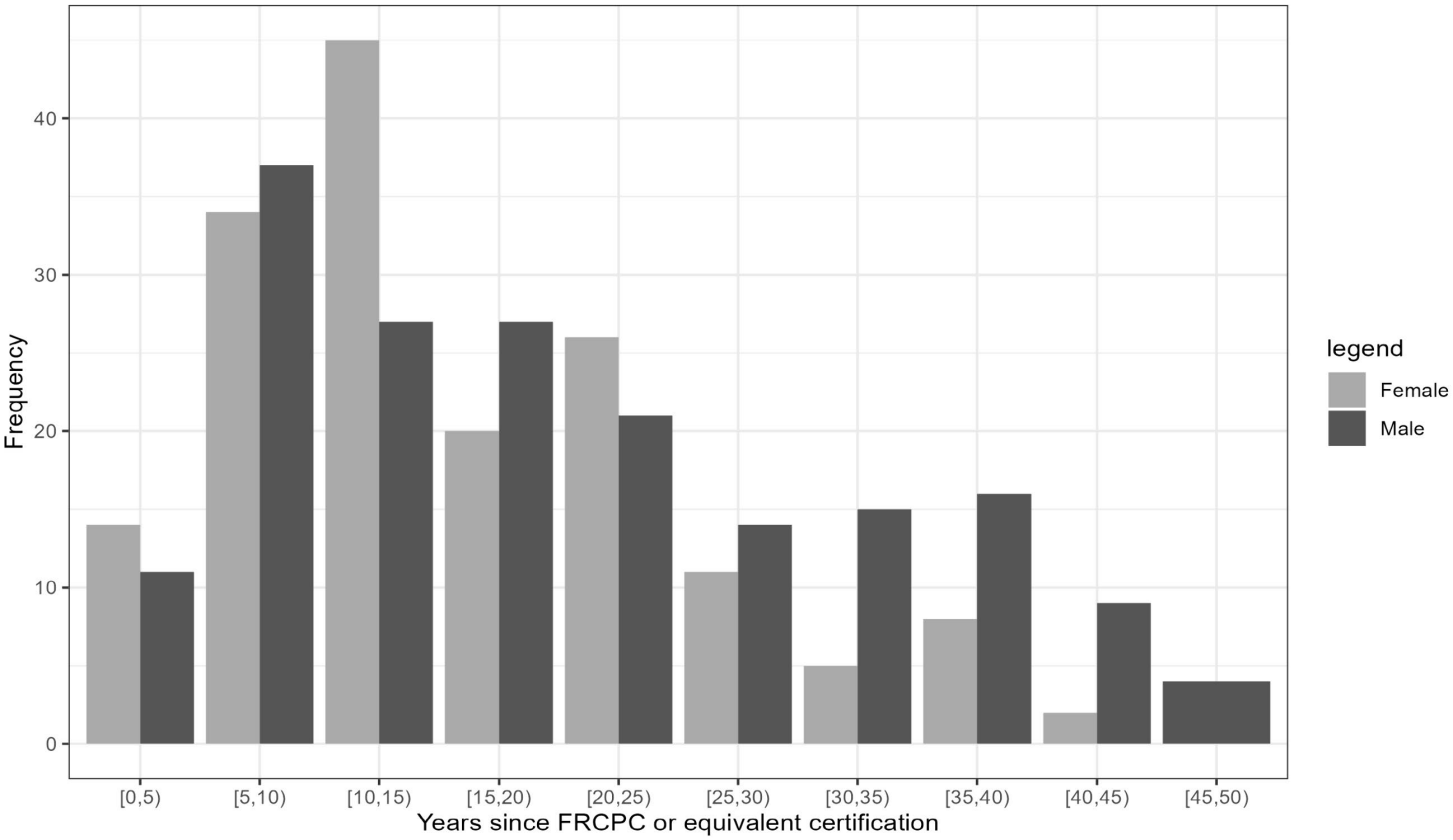
Distribution of years since the Fellow of the Royal College of Physicians and Surgeons of Canada (FRCPC) or equivalent certification among females and males.

**Figure 2:**
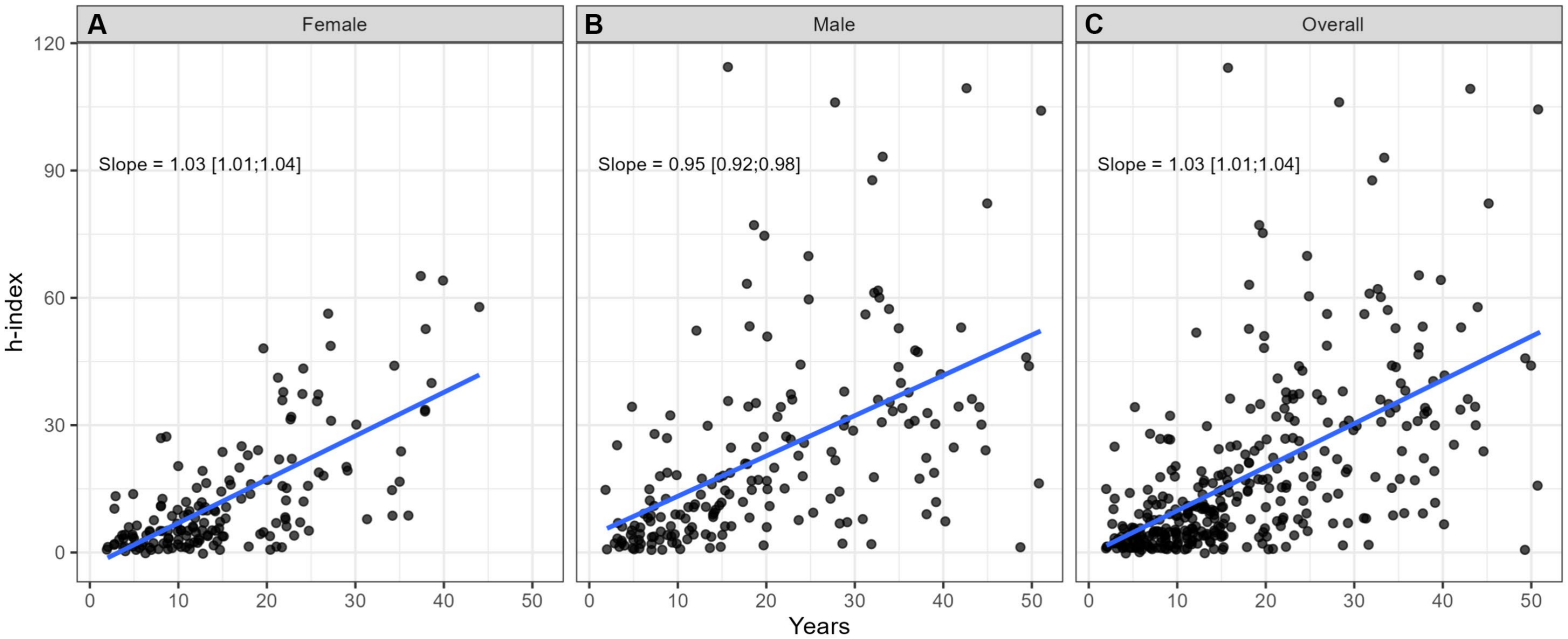
Impact of years since the Fellow of the Royal College of Physicians and Surgeons of Canada (FRCPC) certification or equivalent on h-index for **(A)** females (*n* = 165), **(B)** males (*n* = 184), and **(C)** overall for both sexes (*n* = 349).

**Figure 3:**
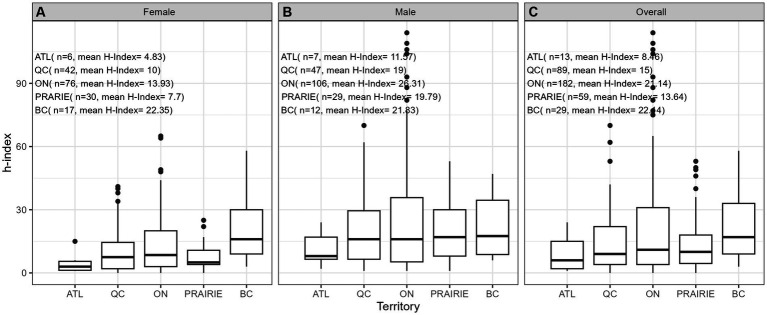
Values of h-index for **(A)** females (*n* = 171), **(B)** males (*n* = 201), and **(C)** overall (*n* = 372) by geographic region.

**Figure 4:**
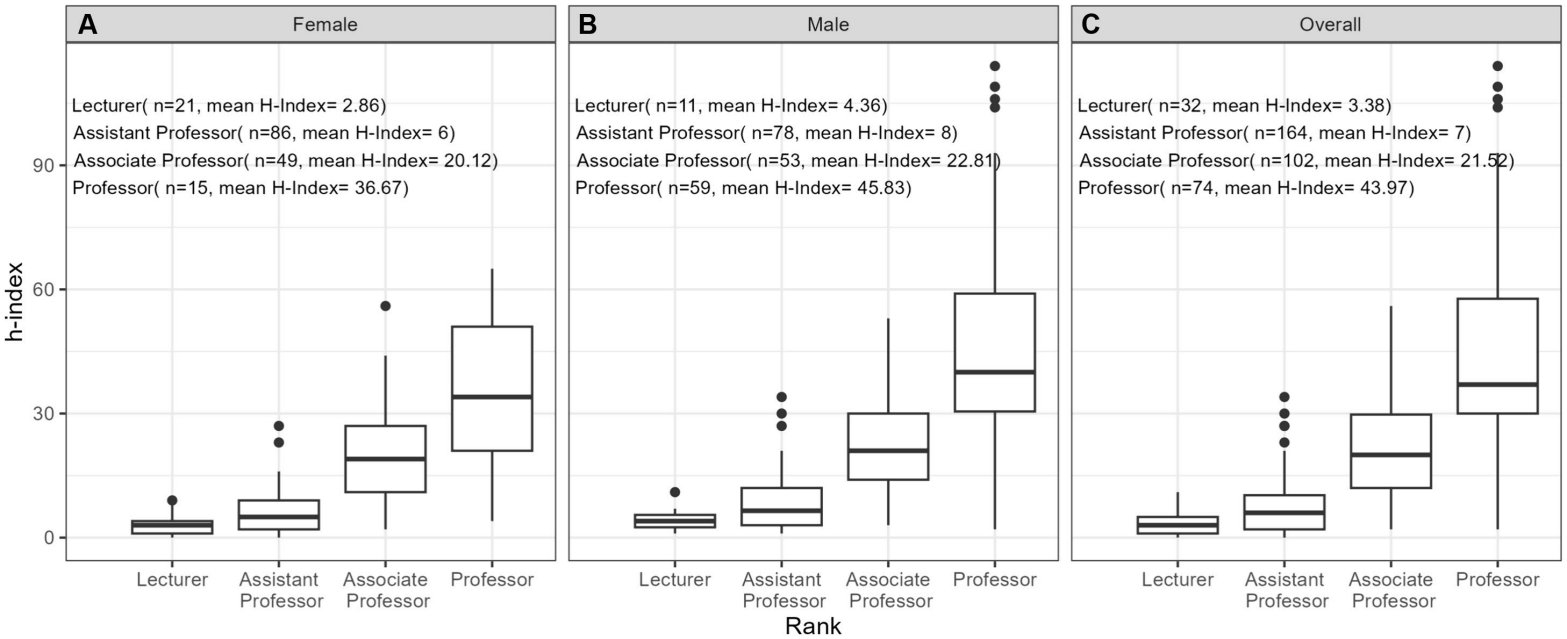
Values of h-index for **(A)** females (*n* = 171), **(B)** males (*n* = 201), and **(C)** overall (*n* = 372) by academic rank.

**Figure 5:**
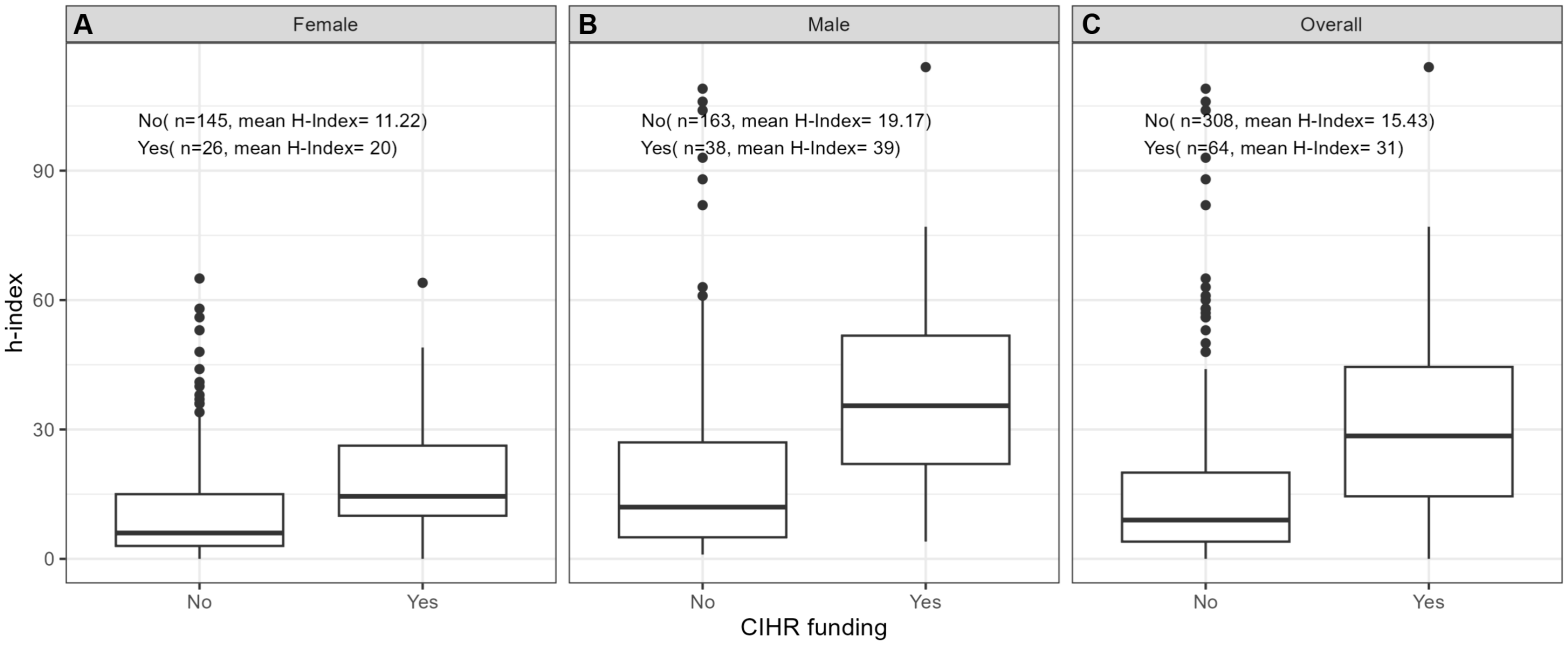
Impact of attainment of Canadian Institutes of Health Research (CIHR) funding on h-index for **(A)** females (*n* = 171), **(B)** males (*n* = 201), and **(C)** overall for both sexes (*n* = 372).

**Figure 6:**
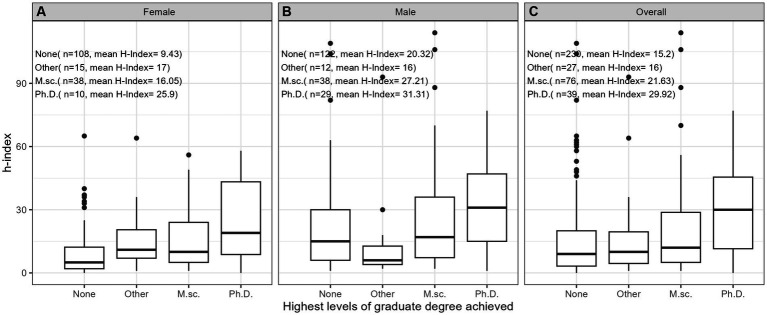
Impact of attainment of graduate degree on h-index **(A)** among females (*n* = 171), **(B)** among males (*n* = 201), and **(C)** overall for both sexes (*n* = 372).

**Figure 7:**
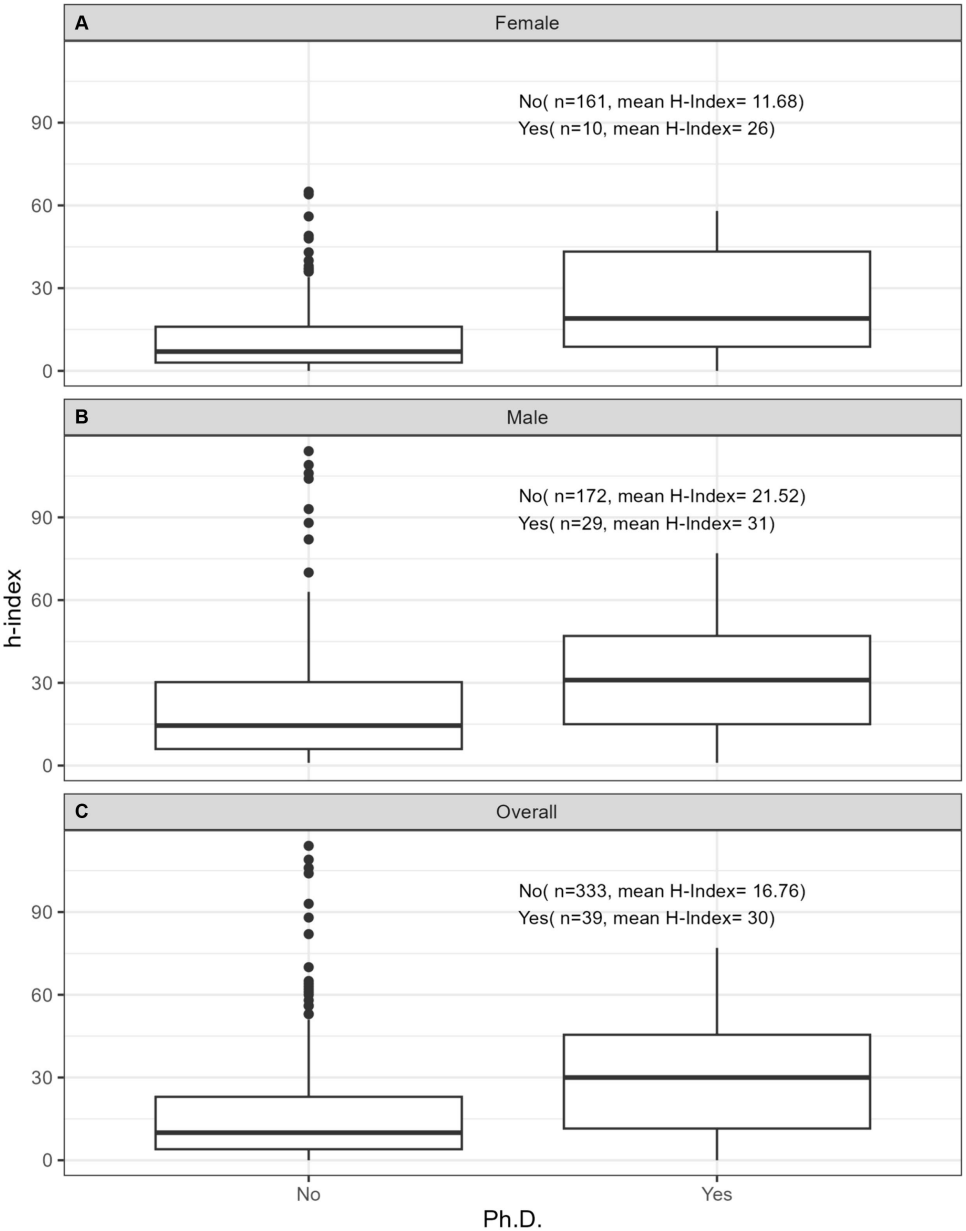
Impact of attainment of Ph.D. degree on h-index for **(A)** females, **(B)** males, and **(C)** overall for both sexes.

Table 2 provides comprehensive descriptive statistics for the cohort divided by sex. The median number of years since FRCPC certification was significantly (*p* = 0.002) lower in females (13.0 [9.00;22.0]) than in males (18.0 [9.0;30.2]). The distribution of ranks was significantly (*p* < 0.001) different among sexes, with females dominating the lower ranks (21 female lecturers vs. 11 males) and males outnumbering in the higher ranks (15 female professors vs. 59 males). We also observed variability by sex in the distribution of years since obtaining the FRCPC, where females constituted the majority of investigators in the first 15 years and males dominated beyond 30 years post-certification. The median h-index in females (8.00 [3.00;16.0]) was significantly lower (*p* < 0.001) than the median h-index in males (16.0 [6.00;33.0]). Figures 1–7 visually depict the associations between different variables and the h-index. When comparing different geographic regions, the median h-index for males was consistently higher than that for females. The rate of increase in h-index varied between males and females; while males started with a higher h-index post-FRCPC, females demonstrated a greater annual increase over time. In both males and females, there was a significant increase in h-index associated with higher academic positions in hematology, as well as with the highest graduate degrees attained. The h-index was found to be higher in both male and female hematologists who obtained CIHR funding.

**Table 2:** Hematology faculty members’ characteristics by sex in Canada.

	Female (%)	Male (%)	Overall	*p*-value
Median number of years since FRCPC certification [IQR]	13.0 [9.00; 22.0]	18.0 [9.0; 30.2]	15.0 [9.0, 24.0]	**0.002**
Graduate degree				**0.044**
None	108 (63)	122 (61)	230 (62)	
Other master degree	15 (9)	12 (6)	27 (7)	
M.Sc.	38 (22)	38 (19)	76 (20)	
Ph.D.	10 (6)	29 (14)	39 (11)	
Rank				**<0.001**
Lecturer	21 (12)	11 (5)	32 (9)	
Assistant Professor	86 (50)	78 (39)	164 (44)	
Associate Professor	49 (29)	53 (26)	102 (27)	
Professor	15 (9)	59 (29)	74 (20)	
CIHR funding				0.421
No	145 (85)	163 (81)	308 (83)	
Yes	26 (15)	38 (19)	64 (17)	
Median h-index [IQR]	8.00 [3.00; 16.0]	16.0 [6.00; 33.00]	11.0 [4.0, 26.3]	**<0.001**

FRCPC, Fellow of the Royal College of Physicians of Canada or equivalent certification; M.Sc., Master of Science; Ph.D., Doctor of Philosophy; CIHR, Canadian Institutes of Health Research.

Our statistical analysis included 372 hematologists whose h-index data was available. It is important to note that there was a 6.18% rate of missing data for this variable “years since FRCPC certification.” The highest GVIF^(1/(2*Df)) was 1.39 for the variable years, meaning there is no multicollinearity concern among independent variables.

Our findings from univariate log-linear analyses are detailed in Table 3. When examining the influence of geographic location, hematologists based in BC demonstrated a significantly higher h-index (ratio = 1.80, *p* = 0.010) compared to QC. However, this difference was not significant for practitioners in Atlantic region, ON, or prairie provinces when compared to QC. When comparing males to females, there was a significant difference in the h-index, where males had a higher h-index, as indicated by a ratio of 1.76 (*p* < 0.001). Furthermore, we found that an increase in the years since FRCPC certification had a significant positive effect on the h-index, with a ratio of 1.77, as expected (*p* < 0.001). Having an M.Sc. or a Ph.D. degree was also significantly associated with a higher h-index (ratio = 1.52, *p* = 0.003) and (ratio = 2.36, *p* < 0.001), respectively, whereas possessing other graduate degrees did not show an impact on academic productivity. Academic rank played a crucial role, with Assistant Professors (ratio = 1.71, *p* < 0.001), Associate Professors (ratio = 5.31, *p* < 0.001), and Professors (ratio = 10.08, *p* < 0.001) all demonstrating significantly higher h-indices compared to faculty lecturers. Finally, obtaining funding from the CIHR was associated with a substantially higher h-index, with a ratio of 2.39 (*p* < 0.001).

**Table 3:** Results of the log-linear univariate and multivariate analyses.

		Univariable models (unadjusted)				Main multivariable model (complete case analyses)				Pooled multivariable model based on 5 imputed datasets using MICE			
Variable	*n*	Expbeta	Lower	Upper	*p*-value	Expbeta	Lower	Upper	*p*-value	Expbeta	Lower	Upper	*p*-value
Territory	372	–	–	–	–	–	–	–	–	–	–	–	–
QC	89	Reference	Reference	Reference	Reference	Reference	Reference	Reference	Reference	Reference	Reference	Reference	Reference
ALT	13	0.62	0.33	1.17	0.14	0.82	0.57	1.19	0.3	0.89	0.62	1.28	0.53
ON	182	1.21	0.93	1.58	0.16	1.35	1.15	1.59	**<0.001**	1.34	1.14	1.58	**<0.001**
PRAIRIE	59	0.93	0.65	1.32	0.68	1.20	0.97	1.47	0.091	1.17	0.95	1.43	0.13
BC	29	1.80	1.15	2.82	**0.010**	1.73	1.32	2.25	**<0.001**	1.76	1.36	2.29	**<0.001**
Sex	372	–	–	–	–	–	–	–	–	–	–	–	–
Female	171	Reference	Reference	Reference	Reference	Reference	Reference	Reference	Reference	Reference	Reference	Reference	Reference
Male	201	1.76	1.42	2.18	**<0.001**	1.18	1.04	1.35	**0.014**	1.19	1.05	1.36	**0.0083**
Years since FRCPC certification	349	1.77	1.64	1.91	**<0.001**	1.03	1.02	1.04	**<0.001**	1.03	1.02	1.04	**<0.001**
Graduate degree	372	–	–	–	–	–	–	–	–	–	–	–	–
None	230	Reference	Reference	Reference	Reference	Reference	Reference	Reference	Reference	Reference	Reference	Reference	Reference
Other master’s degree	27	1.06	0.70	1.60	0.79	1.02	0.79	1.31	0.9	1.01	0.79	1.30	0.91
M.Sc.	76	1.52	1.16	1.99	**0.0027**	1.32	1.11	1.57	**0.0022**	1.29	1.09	1.54	**0.0034**
Ph.D.	39	2.36	1.60	3.46	**<0.001**	1.47	1.15	1.87	**0.0019**	1.38	1.11	1.71	**0.0042**
Rank	372	–	–	–	–	–	–	–	–	–	–	–	–
Lecturer	32	Reference	Reference	Reference	Reference	Reference	Reference	Reference	Reference	Reference	Reference	Reference	Reference
Assistant Professor	164	1.71	1.31	2.23	**<0.001**	1.71	1.34	2.17	**<0.001**	1.71	1.35	2.18	**<0.001**
Associate Professor	102	5.31	4.02	7.04	**<0.001**	3.53	2.70	4.61	**<0.001**	3.42	2.63	4.44	**<0.001**
Professor	74	10.08	7.51	13.52	**<0.001**	4.76	3.49	6.50	**<0.001**	4.63	3.42	6.28	**<0.001**
CHIR funding	372	–	–	–	–	–	–	–	–	–	–	–	–
No	308	Reference	Reference	Reference	Reference	Reference	Reference	Reference	Reference	Reference	Reference	Reference	Reference
Yes	64	2.39	1.81	3.17	**<0.001**	1.38	1.14	1.67	**0.001**	1.40	1.17	1.68	**<0.001**

Statistical significance is denoted by *p* values. MICE, multivariate imputation by chained equations; ATL, Atlanti region; QC, Quebec; ON, Ontario; PRAIRIE, Prairie provinces; BC, British Columbia; FRCPC, Fellow of the Royal College of Physicians of Canada or equivalent certification; M.Sc., Master of Science; Ph.D., Doctor of Philosophy; CIHR, Canadian Institutes of Health Research.

The outcomes of our multivariate analyses, as detailed in Table 3, offer a more nuanced understanding of the factors influencing the h-index among academic hematologists. When considering all independent variables collectively, several factors emerged as significant contributors to a higher h-index, including the time since obtaining FRCPC certification (ratio 1.03, *p* < 0.001) and receiving CIHR funding (ratio 1.38, *p* = 0.001). The geographical aspect also played a role, with hematologists practicing in BC (ratio = 1.73, *p* < 0.001) or ON (ratio = 1.35, *p* < 0.001) demonstrating notably superior h-indices compared to their counterparts in QC, Atlantic and prairie provinces. Furthermore, all levels of academic rank demonstrated noteworthy correlations with higher h-index compared to faculty lecturers, underscoring the influence of academic seniority on research output and *vice-versa*. Male sex was a predictor of having a higher h-index (ratio = 1.18, *p* = 0.014). Additionally, holding a Ph.D. or an M.Sc. degree did substantially impact academic productivity compared to those only holding an M.D. degree (ratio = 1.32, *p* = 0.002) (ratio = 1.47, *p* = 0.002).

To ensure the robustness, we conducted sensitivity analyses, utilizing imputed data for the independent variables. We obtained similar results as in our multivariate analysis, with years since FRCPC certification (ratio = 1.03, *p* < 0.001), CHIR funding (ratio = 1.40, *p* < 0.001), higher academic ranks (assistant professor: ratio = 1.71, *p* < 0.001; associate professor: ratio = 3.42, *p* < 0.001; professor: ratio = 4.63, *p* < 0.001), male sex (ratio = 1.19, *p* = 0.0083), and the obtention of a Ph.D. (ratio = 1.29, *p* = 0.003) or an M.Sc. (ratio = 1.38, *p* = 0.004) independently correlating with a higher h-index. We also found that practicing in ON (ratio = 1.34, *p* < 0.001) or BC (1.76, *p* < 0.001) correlated with a higher h-index compared to working in QC. These sensitivity analyses further support the reliability of our findings.

## Discussion

Our results detail the h-index “curve” in academic hematology in Canada helping individuals correlate their performance with national/regional trends. We identified several factors associated with increased scientific productivity among Canadian academic hematologists. A greater h-index was associated with a longer time since hematology certification, a higher academic rank, certain Canadian jurisdictions (ON and BC), recent CIHR funding, male sex, and attainment of a graduate degree when adjusted for other variables.

Our findings align with our previous research in Canadian academic dermatology (6). This study highlighted a correlation between a higher h-index and specific factors such as years since dermatology certification, successive academic rank, recent research funding from CIHR and attainment a graduate degree. Interestingly, sex was associated with a higher h-index in academic hematology, in contrast to academic dermatology, where while a similar difference was observed favoring male sex, it was not statistically significant.

Similarly, Zaorsky et al.’ s systematic review encompassing various medical fields reported a consistent pattern of an increasing h-index with higher academic ranks (8). Other studies present the same trend in both medical specialties such as radiation oncology (9), dermatology (6, 10, 11), interventional pulmonology (12) and surgical subspecialties including orthopedics (13, 14), surgical oncology (15), and plastic surgery (16).

Sex differences have been reported in gastroenterology (17) and dermatology (11), highlighting that, on average, males had a higher h-index than females. However, these analyses were not conducted while controlling for potential confounding variables and, therefore, could be misleading. Another multivariate analysis also demonstrated sex differences for h-index outcomes in academia, such as in radiation oncology (9). However, the authors did not consider the number of years since certification. Our study found that male hematologists have worked on average longer than female hematologists since their FRCPC certification, which potentially contributed to the sex differences identified in univariate and multivariate analyses. It is important to note that due to lack of data, our study was unable to incorporate information about sick/parental or personal leaves into analysis, which may have impact sexes differentially.

Patel et al. reported females underrepresentation on editorial boards among journals in English language with the highest impact factor in medical oncology, hematology, surgical oncology, and radiation oncology (18). Similarly, Hofstädter-Thalmann identified that female oncologists were less represented in leadership roles despite the increased proportion of females in medicine in Europe (19). Riaz et al. conducted a study evaluating gender disparities in academia among oncologists and hematologists in the United States, finding males held higher academic positions and h-indices, but sex did not affect achieving professorships or leadership roles after adjusting for variables (20). Efforts have been underway to address gender disparities in medicine, and studies have shown that medical school enrollment now has a higher proportion of females than males (21), and that the sex/gender gap is expected to close as more females are advancing in their careers and males are retiring (22).

Riaz et al. reported a median h-index of 11 (IQR = 23) among for the cohort of hematologists and oncologists in the United States (20), which aligns with the findings of our study. Compared to other disciplines, reports show that interventional pulmonologists from Canada and the United States had a median h-index of 2 (mean: 2.53) (12), whereas Canadian dermatologists had a median h-index of 4.0 (IQR:2.00–10.00) (6). American gastroenterologists had a median h-index of 6 (0–99) (17). Among surgical specialties in the US, the median h-index for orthopedics was 5 (IQR:1–12) (13), 7 (0–65) (16) for surgical oncology and 17 for plastic surgery (Range: 1–111) (15).

Despite having almost 25% of all hematologists in Canada and having the second highest median years since FRCPC certification (20.0 [12.0;29.0]), the median h-index in QC was significantly lower than in BC and ON when adjusting for other variables. This is an notable finding that requires further investigation. Although the reasons for the observed differences are not known, our findings could be related to different levels of support available in different provinces for researchers/clinicians across Canada.

There are important limitations to the use of h-index as a measure of academic productivity. The number of papers and the citations the paper receives tend to increase over the years, making the h-index dependent on the individual’s academic career time (23). Furthermore, the h-index cannot decrease and can be misleading when performance changes occur (24). Also, the h-index does not consider self-citation, being susceptible to manipulations and artificial elevation of an author’s h-index (25). In addition, technical limitations, such as having the complete output of scientists with prevalent names, might impact h-index (26). Finally, language of publication can impact the h-index. An analysis by Di Bitetti et al. found that articles published in English have a higher number of citations than those published in other languages, when adjusting for the effect of journal, year of publication and paper length (27). Rovira et al. employed reverse engineering techniques to determine whether the language is a positioning factor in the Google Scholar relevance ranking algorithm (28). Their findings suggest that articles published in languages other than English are significantly lower in the results algorithm, reducing their visibility.

In response to these limitations, the San Francisco Declaration on Research Assessment (DORA) was developed in 2012. It recommends not to rely on metrics, such as h-index as a surrogate measure to assess an individual’s scientific contributions or in hiring, promotion or funding decisions. DORA recommends that the value and impact of all research outputs in addition to publications should be considered when assessing scientific productivity (29).

Despite our efforts in searching multiple platforms, one limitation encountered in our study pertains to missing data for the h-index, academic rank, graduate degree and the number of years since hematology certification and leaves of absence a researcher might have taken. However, we conducted sensitivity analyses for the independent variables, which yielded results comparable to those obtained from the complete data set. The unavailability of Scopus author profiles may be attributed to several factors, including the absence of peer-reviewed publications under a particular name, variations in authors’ names or surnames, or lack of recent publications in the field of hematology, making it challenging to distinguish individuals with similar author names.

Our dataset only included sex information without accounting for gender diversity. Additionally, we relied on online faculty listings, which not might reflect real-time information. We attempted to mitigate this limitation by screening for non-practicing hematologists and excluding them from our cohort. Despite these efforts, there is a possibility that some very recently appointed faculty members might not have been included. Lastly, we lack data regarding each individual’s ethnic background, allocation of research/academic protected time and the level of institutional support, which can influence the h-index. To gain a more comprehensive understanding of academic productivity in hematology, future assessments should consider validated questionnaires and qualitative research methods, enabling a longitudinal examination of the findings reported in our study. Nevertheless, we believe that our study offers a valuable overview of h-index as a measure of academic productivity in hematology in Canada.

## Conclusion

In summary, our study reports the curve for academic hematology in Canada. It highlights that a higher h-index was linked to more years since hematology certification, a higher academic rank, distinct Canadian regions, recent CIHR funding, male sex and attainment of a graduate degree. Longitudinal assessments of academic productivity in hematology in Canada are needed while continuing all efforts to ensure equal opportunities and representation in the field.

## Data Availability

The raw data supporting the conclusions of this article will be made available by the authors, without undue reservation.
